# Long noncoding RNA ZEB1-AS1 acts as a Sponge of miR-141-3p to Inhibit Cell Proliferation in Colorectal Cancer

**DOI:** 10.7150/ijms.46698

**Published:** 2020-06-27

**Authors:** Guanghai Wu, Mei Xue, Yongjie Zhao, Youkui Han, Chao Li, Shuai Zhang, Judong Zhang, Jing Xu

**Affiliations:** 1 Department of General Surgery, Tianjin Union Medical Center, Jieyuan Road 190, Hongqiao District, Tianjin, 300121, China.; 2 NHC Key Laboratory of Hormones and Development, Tianjin Key Laboratory of Metabolic Diseases, Chu Hsien-I Memorial Hospital & Tianjin Institute of Endocrinology, Tianjin Medical University, Tianjin 300134, China.

**Keywords:** Colorectal cancer, Long noncoding RNAs, MicroRNAs, Proliferation

## Abstract

Evidence shows that long noncoding RNAs (lncRNAs) play key roles in various cancers, including colorectal cancer. In this current study, we found that the expression of ZEB1-AS1 in colorectal cancer tissues and cell lines was significantly upregulated, and positively correlated with advanced stage of colorectal cancer. Kaplan-Meier assays also indicated that the expression of ZEB1-AS1 was correlated with poor prognosis in patients with colorectal cancer. Knocking down of ZEB1-AS1 inhibited the proliferation of colorectal cancer cells. Subcellular fractionation analyses suggested that ZEB1-AS1 was majorly distributed in cytoplasm of SW480 and LOVO cells. Thus, ZEB1-AS1 might act as a competing endogenous RNA. MicroRNA array analysis suggested that miR-141-3p was significantly downregulated in CRC tissues, which was further verified by RT-qPCR. The results of luciferase reporter assay proved that miR-141-3p was a target of ZEB1-AS1. Functionally, miR-141-3p inhibitor reversed the anti-proliferation effect of sh-ZEB1-AS1 on colorectal cancer cells. Collectively, ZEB1-AS1 may contribute to colorectal cancer cell proliferation by sponging miR-141-3p.

## Introduction

Colorectal cancer (CRC) is the third most common cancer worldwide and corresponds to the second cause of death from cancer, with more than 1 million new cases per year 1. Although the mortality from colorectal cancer has decreased under the latest advances in treatment and early detection, however, the death rate of CRC is still about 35% 2. In addition, there are still more than 50% of patients with CRC that died from distant metastasis 3. In spite lots of efforts have been made to understanding the pathophysiology of CRC. However, the related mechanisms are elusive and remain unknown. Clarification of the related mechanisms involved in tumor metastasis and progression may help of the diagnosis and treatments, which will further improve the clinical outcome.

Long noncoding RNAs (lncRNAs) refer to transcribed RNA molecules that are over 200 nucleotides but lack significant protein-coding potential 4, 5. Growing evidences have shown that lncRNAs participant in various biological processes, such as glycolytic reprogramming 6 and repair, vasculo-genesis, miRNAs silencing, apoptosis 7 and epithelial-mesenchymal transition (EMT) 8. LncRNAs are important regulators in various human cancers 9-11, by regulating proliferation, differentiation, apoptosis, and metastasis of cancer cells 12, 13. LNCAROD promotes cancer progression via forming a ternary complex with HSPA1A and YBX1 in head and neck squamous cell carcinoma 13. LncRNA CMPK2 promotes colorectal cancer progression by activating the FUBP3-c-Myc axis 14. LncRNA SLC2A1-AS1 regulates aerobic glycolysis and progression in hepatocellular carcinoma via the STAT3/FOXM1/GLUT1 pathway 15. LncRNAs act as competing endogenous RNAs (ceRNAs) modulating the expression of microRNAs (miRNAs) in a cell-type dependent manner 16. LncRNAs decrease miRNA levels and lead to the increase in the expression of miRNA target genes 16, 17. LncRNA ZEB1-AS1 has been reported to play a key role in various types of human cancers, especially the digestive system neoplasm 18-21. Study showed that lncRNA ZEB1-AS1 may promote colon adenocarcinoma malignant progression via miR-455-3p/PAK2 axis 22. However, the specific role of ZEB1-AS1 in CRC has not been fully clarified.

In this current study, we reported that the expression of ZEB1-AS1 was upregulated in CRC tissues compared to that in the corresponding adjacent normal tissues. Silence of ZEB1-AS1 suppressed the proliferation of CRC cells. The results delineate that ZEB1-AS1 promotes CRC cell proliferation through sponging miR-141-3p. Therefore, our study will provide new insights into the molecular function of the ZEB1-AS1/miR-141-3p axis in CRC and highlight the potential of lncRNAs to act as new therapeutic targets.

## Materials and Methods

### Clinical samples

27 CRC tissues and adjacent normal tissues were obtained from Tianjin Union Medical Center. Patients were eligible for the study if (1) they did not receive any chemotherapy or radiotherapy before the surgery; (2) surgical specimens of tumor lesions were available. This process was approved by Tianjin Union Medical Center and written informed consents were obtained from the involved patients. The tissues were quickly placed in liquid nitrogen for RNA testing.

### Cell culture

Four human CRC cell lines (SW480, LOVO, HT29 and PKO) and a normal human colon epithelial cell line NCM460 were purchased from the cell bank of the Chinese Academy of Sciences (Shanghai, China). Cells were cultured in RPMI 1640 medium (Gibco, Carlsbad, CA, USA), which were supplemented with 10% fetal bovine serum (FBS, Gibco, NY, USA) at a condition of 37 °C with a humidified atmosphere containing 5% CO_2_.

### Transfection

Specific shRNAs against ZEB1-AS1 (sh-ZEB1-AS1#1 and sh-ZEB1-AS1#2) and corresponding NCs (sh-NCs) were acquired from RiboBio (Guangzhou, China). Moreover, miR-141-3p mimics, miR-141-3p inhibitors, NC mimics and NC inhibitors were acquired from GenePharma (Shanghai, China). SW480 or LOVO cells were transfected with these plasmids through Lipofectamine 3000 (Invitrogen, CA, USA), separately.

### Quantitative real time polymerase chain reaction (RT-qPCR)

RT-qPCR was performed in accordance with the previous study 23. Total RNA from tissues and cells was extracted by TRIzol reagent (Invitrogen) and inversely transcribed into cDNA using a Reverse Transcription Kit (Thermo, USA). The primers used in this study were obtained from Genscript Corp (Nanjing, China). Then RT-qPCR was carried out using SYBR Select Master Mix (Thermo, Waltham, MA, USA). Relative expression was normalized to U6 or GAPDH and all were measured by the comparative Ct (ΔΔ Ct) method.

### CCK-8 assay

CCK-8 assay was conducted by using cells seeded at a density of 1×10^5^/ml into 96-well plates. After 24, 48, 72 and 96 h incubation with different treatments, 10 ml CCK-8 reagents (Qianshang, Wuxi, China) was added to each well. The 450 nm absorbance of the plates was determined by a microplate reader.

### Luciferase reporter assays

ZEB1-AS1-wt and ZEB1-AS1-mut sequence were inserted into pMIR-reporter (Promega, USA). The pmirGLO-ZEB1-AS1-wt/mut was co-transfected into SW480 or LOVO cells with mimics NC or miR-141-3p mimics. Dual-Luciferase Reporter Assay System (Promega, Madison, WI, USA) was employed for measuring luciferase activity.

### Gene Chip Technology Analysis

Total RNA was isolated using Trizol (Invitrogen, USA) and purified with an RNeasy mini kit (Omega, USA) according to the manufacturer's instructions. Shanghai Biotechnology Co., Ltd conducted the microRNA microarray gene expression experiments and data analysis.

### Subcellular fractionation

Nuclear or cytoplasmic RNA in cells were separated and purified by a Cytoplasmic and Nuclear RNA Purification Kit (Norgen Biotek, Thorold, Canada). Expression of ZEB1-AS1, GAPDH and U6 in nuclear and cytoplasm fractions from SW480 or LOVO cells was determined via RT-qPCR, respectively.

### Cell cycle analysis

Cell cycle analysis was performed using flow cytometry. After 48 hours of treatment, the cells were harvested and washed twice with cold phosphate buffer saline buffer, and then fixed with 75% alcohol for 12 h at 4 °C. After washing, cells were treated with RNase (50 µg/mL) and stained with propidium iodide (50 µg/mL) at 4 °C for 30 min before being analyzed with a BD FACSCalibur flow cytometer (BD Pharmingen, USA). Data was analyzed using Modfit software (Verity Software House, USA).

### Immunofluorescence (IF) assays

Cells in plates were fixed and permeated with 0.3% Triton X-100 at room temperature. Then, cells were blocked with bovine serum album in phosphate-buffered saline for 2 h at room temperature and incubated with primary antibodies for anti-PCNA (1:1000, Proteintech, USA), anti-Ki67 (1:100, Proteintech, USA) at 4 °C overnight. After three times washing, cells were incubated with secondary antibody. DAPI was added to the samples to visualize cell nuclei. The staining was captured by a Leica DMI 4000 B automated inverted microscope equipped with a Leica DFC300 FX camera.

### Statistical analysis

SPSS 19.0 software was used for statistical analysis. All the values were expressed as mean ± Standard Deviation (SD). Data was analyzed using one-way analysis of variance (ANOVA) or the LSD-test. Pearson correlation test was conducted to measure the association among ZEB1-AS1, and miR-141-3p expression. A value of *P* < 0.05 was considered statistically significant.

## Results

### ZEB1-AS1 is upregulated and associated with poor prognosis in CRC

**Figure 1:**
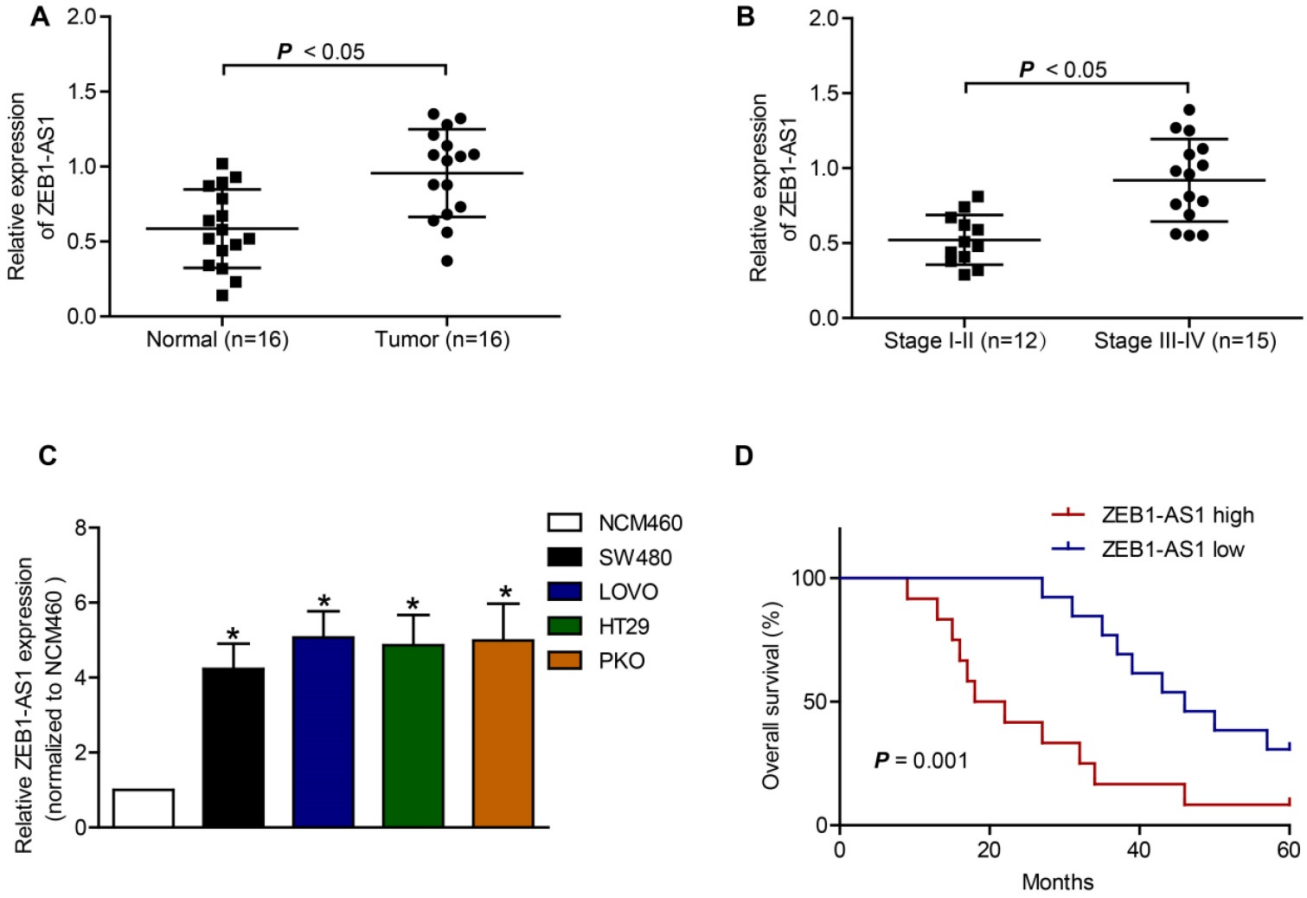
Upregulation of ZEB1 is associated with poor prognosis in CRC. (**A**) ZEB1-AS1 expression was detected by RT-qPCR in normal and tumor tissues. (**B**) RT-qPCR analysis of ZEB1-AS1 expression in stage I-II CRC tissues and stage III-IV CRC tissues. (**C**) ZEB1-AS1 was upregulated in CRC cell lines SW480, LOVO, PKO and HT29, as comparing with that of a normal human colon epithelial cell line NCM460. (**D**) Kaplan-Meier survival analyses indicated that upregulation of ZEB1-AS1 in CRC patients predicted worse overall survival. *P < 0.05.

The basic characteristics of the patients included were listed in Table 1. RT-qPCR was applied to measure the expression of ZEB1-AS1 in CRC tissue samples and CRC cell lines. The expression of ZEB1-AS1 was significantly increased in CRC tissues, compared to that in the neighboring normal tissues (Figure 1A). The results also showed that the upregulation of ZEB1-AS1 was positively correlated with advanced stage of CRC (Figure 1B). Further analysis indicated that the ZEB1-AS1 expression was significantly upregulated in CRC cell lines (Figure 1C). Kaplan-Meier assays indicated that the CRC patients with higher ZEB1-AS1 expression displayed poorer overall survival than those with lower expression (Figure 1D). Collectively, the expression of ZEB1-AS1 was increased in CRC tissues and cell lines and correlated with poor prognosis in CRC patients.

### Downregulation of ZEB1-AS1 inhibited proliferation of colorectal cancer cells

**Figure 2:**
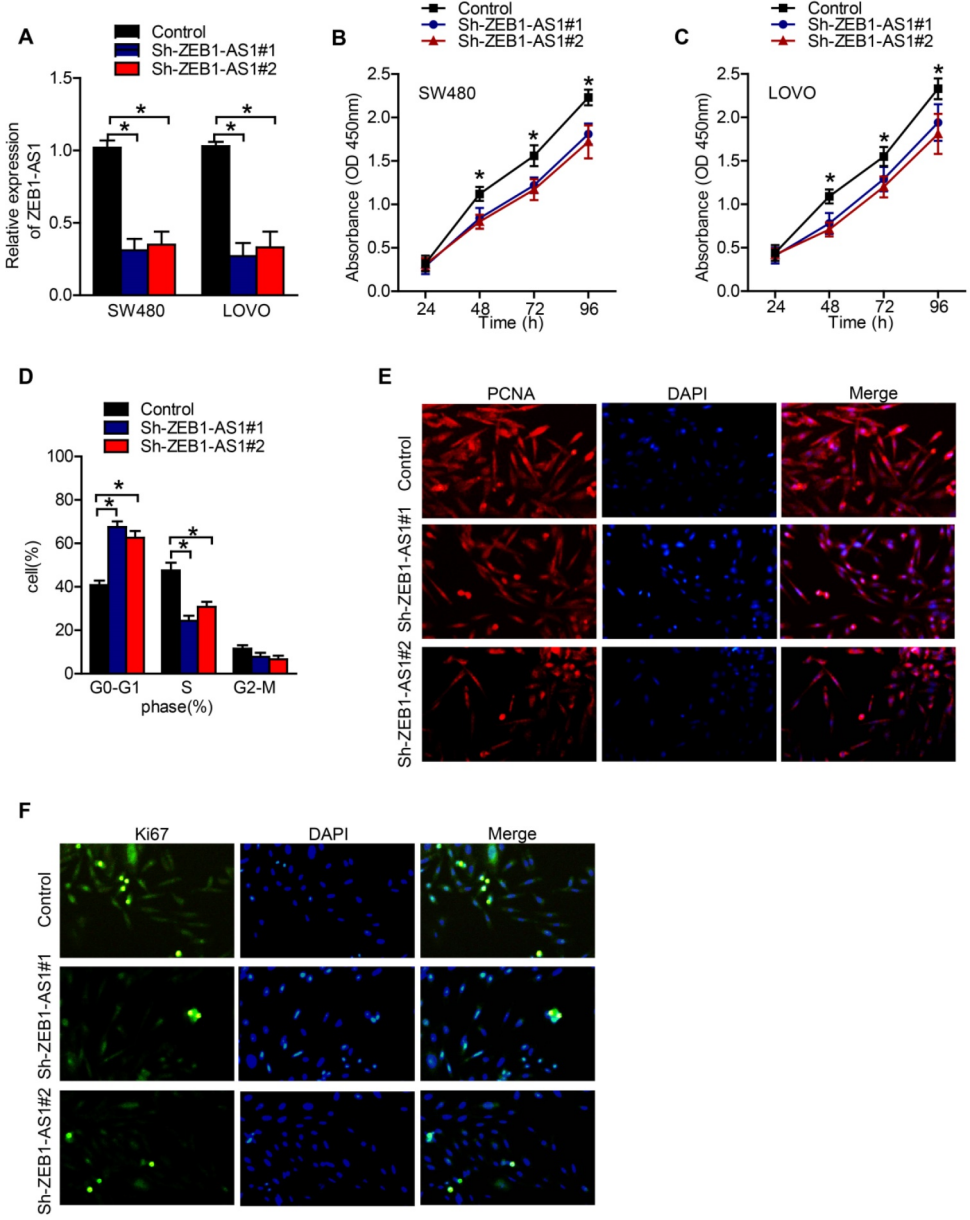
ZEB1-AS1 promotes cell proliferation in CRC cells. (**A**) RT-qPCR analysis of ZEB1-AS1 expression in SW480 and LOVO. (**B-C**) CCK8 assay was conducted to test cell proliferation in SW480 and LOVO cells. (**D**) Flow cytometry analysis of cell cycle in SW480 treated for 48 h. (**E-F**) Immunofluorescence staining of PCNA and Ki67 in SW480 treated for 48 h. *P < 0.05.

Specific shRNAs were used to suppress the expression of ZEB1-AS1 in CRC cells. RT-qPCR showed that ZEB1-AS1 expression was knocked down in SW480 and LOVO cells successfully (Figure 2A). The results of CCK-8 assay showed that knockdown of ZEB1-AS1 inhibited the proliferation of SW480 and LOVO cells (Figure 2B and C). The patterns of cell cycle distribution in SW480 were detected by flow cytometry. As the data showed, compared with negative control, silence of ZEB1-AS1 induced an increment of cell proportion in G0-G1 phase, and a decrement in S phase and G2-M phase (Figure 2D). The expression of PCNA and Ki67, markers of proliferating cells, were detected by IF. As our results showed that, both PCNA and Ki67 were suppressed by the silence of ZEB1-AS1 (Figure 2E and F). Collectively, the above results indicated that ZEB1-AS1 might promote CRC by facilitating cancer cell proliferation.

### ZEB1-AS1 sponges miR-141-3p in CRC cells

**Figure 3:**
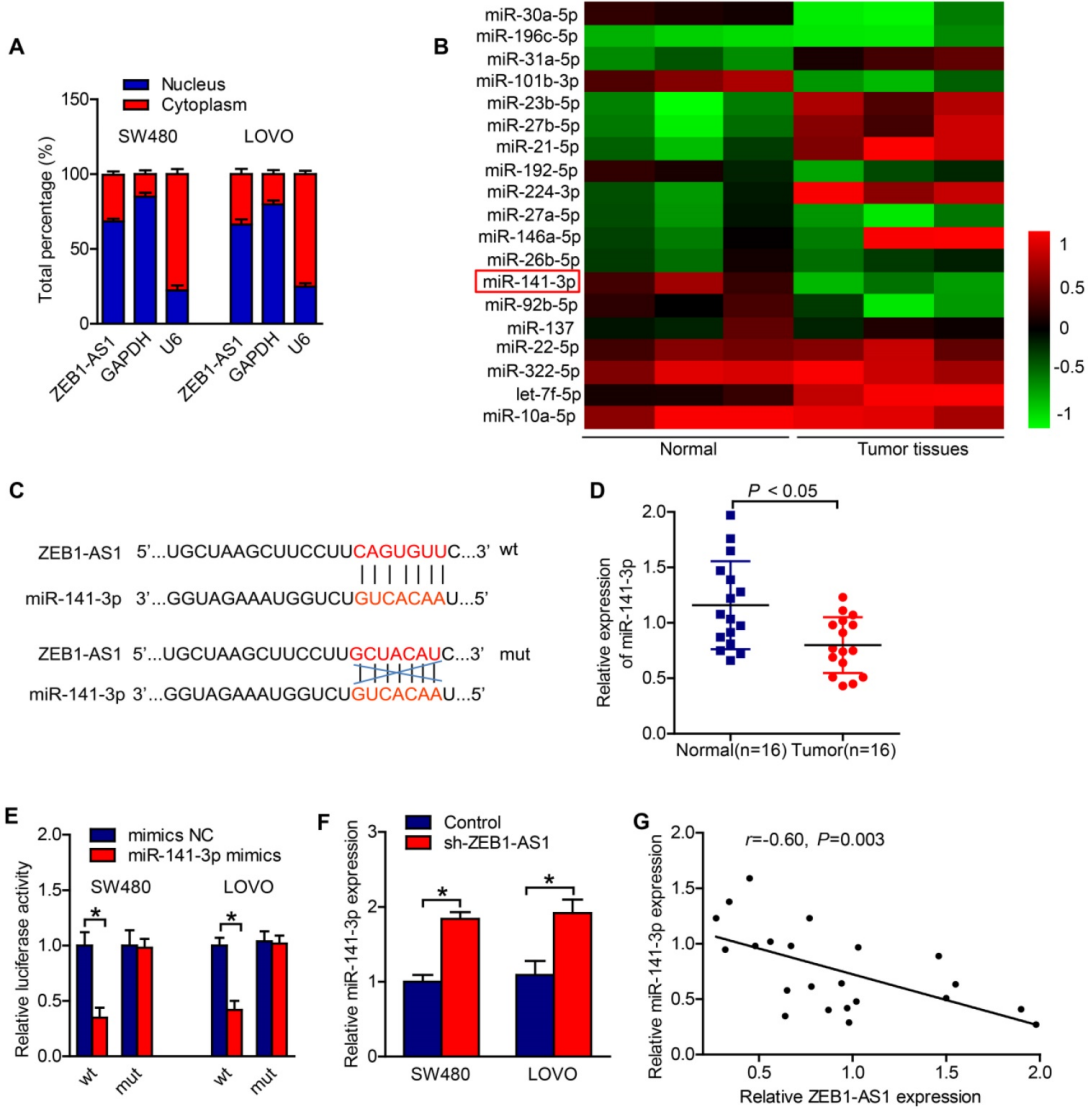
ZEB1-AS1 sponges miR-141-3p in CRC cells. (**A**) Nuclear and cytoplasmic fractionation was analyzed for ZEB1-AS1 expression in SW480 and LOVO. (**B**) The microRNA array analysis in normal and tumor tissues. (**C**) The potential binding sites between ZEB1-AS1 and miR-141-3p. (**D**) The expressions of miR-141-3p in CRC tissues were detected by RT-qPCR. (**E**) Luciferase reporter assay showed ZEB1-AS1-wt activity was impaired by miR-141-3p. (**F**) The expression of miR-141-3p in SW480 and LOVO was upregulated after ZEB1-AS1 expression was downregulated identified by RT-qPCR. (**G**) The expression of miR-141-3p was negatively correlated with ZEB1-AS1 expression in CRC tissues. *P < 0.05.

To further decipher the potential mechanism whereby ZEB1-AS1 regulates the CRC progress, we adopted subcellular fractionation to detect the localization of ZEB1-AS1 in cytoplasm and nucleus of CRC cells. The result suggested that ZEB1-AS1 was majorly distributed in cytoplasm of SW480 and LOVO cells (Figure 3A). Thus, we speculated that ZEB1-AS1 might be a ceRNA in colorectal tumor progression. Our previous microRNA array analysis suggested that miR-141-3p was significantly downregulated in CRC tissues, as compared with vehicle treatment (Figure 3B). And we found that miR-141-3p was a potential target of ZEB1-AS1 by searching starBase (Figure 3C). RT-qPCR results displayed that the expression of miR-141-3p in CRC tissues was relatively lower than that in the neighboring normal tissues (Figure 3D). The interaction between miR-141-3p and ZEB1-AS1 was examined by luciferase reporter assay. The results in our study showed that miR-141-3p decreased the luciferase activity of ZEB1-AS1-wt reporter's, but had no effect on the ZEB1-AS1-mut reporter's luciferase activity (Figure 3E).

Moreover, the expression of miR-141-3p in CRC cells was upregulated after the expression of ZEB1-AS1 was suppressed (Figure 3F). The results of Pearson correlation analysis showed that the expression of ZEB1-AS1 was negatively correlated with miR-141-3p expression in CRC tissues (Figure 3G). The above results suggested that ZEB1-AS1 sponges miR-141-3p in CRC cells.

### ZEB1-AS1 contributes to CRC cell proliferation via sponging miR-141-3p

**Figure 4:**
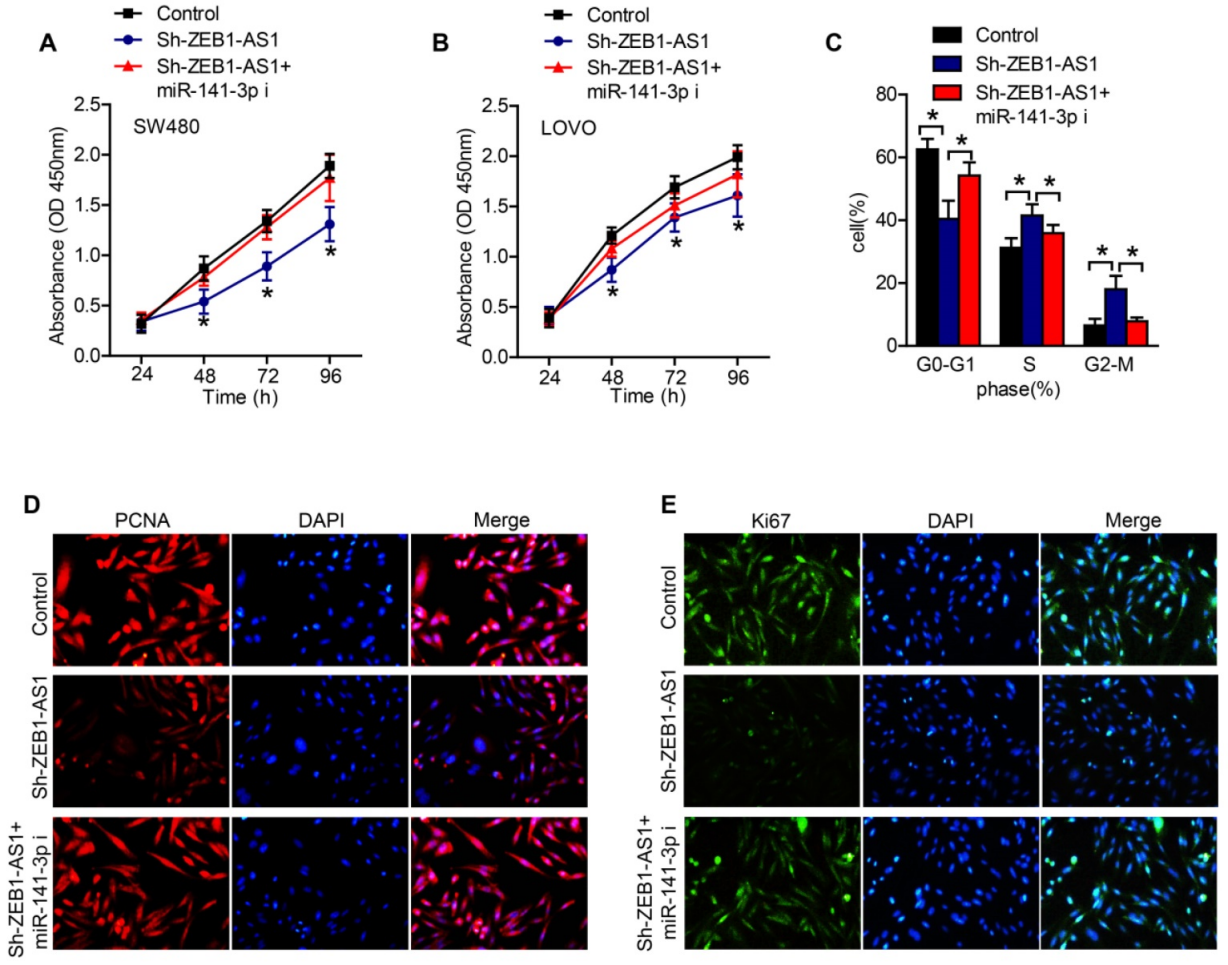
ZEB1-AS1 contributes to CRC cell proliferation via sponging miR-141-3p. (**A and B**) CCK-8 was applied to test the effect of miR-141-3p inhibitor on cell proliferation suppressed by sh-ZEB1-AS1 in SW480 and LOVO. (**C**) Flow cytometry analysis of cell cycle in SW480 treated for 48 h. (**E-F**) Immunofluorescence staining of PCNA and Ki67 in SW480 treated for 48 h. *P < 0.05. miR-141-3p i: miR-141-3p inhibitor.

In order to certify whether ZEB1-AS1 promote cancer cell proliferation via sponging miR-141-3p in CRC cells, we conducted a series of functional studies. CCK-8 assays revealed that the silence of ZEB1-AS1 obviously suppressed the proliferation of CRC cells, while inhibition of miR-141-3p abolished the effect of sh-ZEB1-AS1 on the proliferation of CRC cells (Figure 4A and B). The results of flow cytometry showed that, silence of ZEB1-AS1 induced an increment of cell proportion in G0-G1 phase, and a decrement in S phase and G2-M phase (Figure 4C). However, inhibition of miR-141-3p reversed the role of sh-ZEB1-AS1 (Figure 4C). The staining of PCNA and Ki67 also supported the above results, that miR-141-3p inhibitor reversed the role of sh-ZEB1-AS1 in CRC cells (Figure 4D and E). Collectively, ZEB1-AS1 may promote CRC progression via sponging miR-141-3p.

## Discussion

The identification of novel therapeutic targets has a very essential clinical significance for the improvement of long-term survival of CRC. Growing evidence has suggested that lncRNAs are tightly linked to the initiation and development of human cancers 14, 24-26. Previous studies have suggested a vital role of ZEB1-AS1 in the digestive system neoplasm, such as bladder cancer 19, gastric cancer 20 and liver cancer 21. Studies have reported that various lncRNAs participate in the progression of CRC. LDLRAD4-AS1 promotes metastasis by decreasing the expression of LDLRAD4 and predicts a poor prognosis in colorectal cancer 27. METTL14 suppresses proliferation and metastasis of colorectal cancer by down-regulating oncogenic long non-coding RNA XIST 28. The lncRNA TUG1 is required for TGF-β/TWIST1/EMT-mediated metastasis in colorectal cancer cells 29.

In this current study, we explored the role of lncRNA ZEB1-AS1 in the progression and prognosis of CRC. Although several studies have declared the role of ZEB1-AS1 in CRC, such as ZEB1-AS1 might promote malignant progression via miR-101/ZEB1 axis 30, promote cell proliferation by regulating miR-181a-5p/Wnt/β-catenin signaling 31 and predict progression and poor prognosis of colorectal cancer 32. However, the specific role of ZEB1-AS1 in CRC has not been fully elaborated. In our study, ZEB1-AS1 could promote the proliferation of CRC cells, which was in consistent with previous studies mentioned above. Our results revealed that ZEB1-AS1 promotes and predicts poor prognosis of CRC by targeting at miR-141-3p.

In this study, the expression of ZEB1-AS1 in CRC tissues and CRC cell lines was significantly upregulated, and was positively correlated with advanced stage of CRC. Kaplan-Meier assays also indicated that the expression of ZEB1-AS1 was correlated with poor prognosis in patients with CRC. Subsequently, silence of ZEB1-AS1 inhibited the proliferation of CRC cells, as demonstrated by CCK-8 assay and flow cytometry. The staining of PCNA and Ki67, markers for proliferation, also verified our previous results. Briefly, ZEB1-AS1 works as a cancer-promoting gene in CRC. After confirming the proliferation promotion role of ZEB1-AS1 in CRC, we further exploring the underlying mechanisms.

The ceRNA hypothesis was proposed to describe the function of many lncRNAs as ceRNA to protect the genuine targets of miRNAs from silencing or translational suppression 33. Previous studies demonstrated that miR-205 34, miR-101 30 and miR-181a-5p 31 might be target miRNAs of ZEB1-AS1 in CRC. Our previous microRNA array analysis suggested that miR-141-3p richly expressed in normal colorectal tissues, but was significantly downregulated in CRC tissues. By searching starBase, we found that miR-141-3p was a potential target of ZEB1-AS1. We speculated that the interplay between ZEB1-AS1 and miR-141-3p might well explain the promotion role of ZEB1-AS1 in CRC. The luciferase reporter assay further verified our speculation that miR-141-3p is a target of ZEB1-AS1. MiR-141-3p is a novel miRNA involved in cancer 35-37. Recently studies showed that miR-141-3p might participate in the progression of CRC by directly targeting EGFR 38, TRAF5 39 and ZEB1 40. To further explore the association between ZEB1-AS1 and miR-141-3p, rescued-function test was used, and found that miR-141-3p inhibitor reversed the role of sh-ZEB1-AS1 in CRC cells. Collectively, ZEB1-AS1 may promote CRC progression via sponging miR-141-3p.

In summary, our findings revealed the role of ZEB1-AS1/miR-141-3p axis in the progression of CRC. ZEB1-AS1 may serve as a promising factor to predict prognosis and therapeutic target against CRC.

## Figures and Tables

**Table 1:** Basic information of the patients with CRC

Characteristics	ZEB1-AS1		*P*-value
	Low	High	
**Gender**			n.s.
Male	6	7	
Female	7	7	
**Age**	62.5±3.8	63.8±4.5	n.s.
**Location**			n.s.
Colon	5	7	
Rectal	8	7	
**TNM stage**			<0.05
I-II	8	5	
III-IV	5	9	
**Metastasis**			<0.05
Yes	4	11	
No	9	3	

n.s.: no significance.

## Abbreviations

lncRNAs: long noncoding RNAs
CRC: colorectal cancer
ceRNA: competing endogenous RNA
miRNAs: microRNAs
FBS: fetal bovine serum
EMT: Epithelial to mesenchymal transition
RT-qPCR: Quantitative real time polymerase chain reaction
miR-141-3p i: miR-141-3p inhibitor
